# Hematology and *toll-like receptors 2* and *4* and *lipopolysaccharide-induced tumor necrosis factor-α factor* gene expression in peripheral blood mononuclear cells of Thai indigenous chickens

**DOI:** 10.14202/vetworld.2022.2795-2799

**Published:** 2022-12-07

**Authors:** Chananphat Tantikositruj, Asep Gunawan, Muhammad Jasim Uddin, Wirawan Nuchchanart, Chaiwat Boonkaewwan, Watchara Laenoi, Autchara Kayan

**Affiliations:** 1 Department of Animal Science, Faculty of Agriculture, Kasetsart University, Ngam Wong Wan, Bangkok 10900, Thailand; 2 Department of Animal Production and Technology, Faculty of Animal Science, IPB University, Bogor 16680, Indonesia; 3 School of Veterinary Medicine, Murdoch University, Western Australia 6155, Australia; 4 Department of Animal Science, Faculty of Agriculture at Kamphaeng Saen, Kasetsart University, Kamphaeng Saen Campus, Nakhon Pathom 73140, Thailand; 5 Akkhraratchakumari Veterinary College, Walailak University, Nakhon Si Thammarat 80161, Thailand; 6 Department of Animal Science, School of Agriculture and Natural Resources, University of Phayao, Phayao 56000, Thailand

**Keywords:** blood hematology, gene expression, immune, Thai indigenous chicken

## Abstract

**Background and Aim:**

Toll-like receptors (TLRs) play crucial roles in the early phase of infection in the innate immune response against bacteria, viruses, fungi, and parasites. Lipopolysaccharide-induced tumor necrosis factor-α factor (LITAF) is an essential transcription factor that regulates the immune system, apoptosis, and inflammatory cytokines. This study aimed to determine the hematological profile reflecting the immune response related to *TLR2* and *TLR4* and *LITAF* gene expression in Thai indigenous chickens.

**Materials and Methods:**

Blood samples (2 mL) were randomly obtained from three chicken breeds (black-boned chicken, Fah Luang chicken, and Pradu Hang Dam chicken) at 16 weeks of age (n = 5 per breed). The hematological profile and mRNA expression within the peripheral blood mononuclear cells (PBMCs) were determined by hematological analysis and quantitative real-time polymerase chain reaction (qRT-PCR).

**Results:**

The hematological profile differed significantly in terms of red blood cells (RBCs), hemoglobin, and white blood cells (WBCs) (p < 0.05). Black-boned chicken and Fah Luang chicken had lower RBC levels than Pradu Hang Dam chicken. Fah Luang chicken had lower hemoglobin than Pradu Hang Dam chicken. However, Fah Luang chicken had higher WBC levels than Pradu Hang Dam chicken. Hematocrit, heterophils, basophils, eosinophils, lymphocytes, and monocytes did not differ significantly among the groups (p > 0.05). According to qRT-PCR, the expression of the *TLR2* gene did not differ significantly among the groups (p > 0.05), while *TLR4* and *LITAF* gene expression did (p < 0.05). *Toll-like recepto*r 4 and *LITAF* genes were most highly expressed in Fah Luang chicken.

**Conclusion:**

The PBMCs of Thai indigenous chickens showed evidence of *TLR4* and *LITAF* gene expression, with higher expression levels observed in Fah Luang chicken. From this preliminary study, it is concluded that *TLR4* and *LITAF* genes might play roles in the main immune system response in Thai indigenous chickens.

## Introduction

Blood metabolites are used to evaluate heat stress levels in animals. The immune system plays specialized roles in defense against infection. Innate (natural) and acquired (adaptive) reactions are two categories of immune responses. Natural killer cells, basophils, mast cells, and eosinophils that release inflammatory mediators, as well as phagocytic cells such as neutrophils, monocytes, and macrophages, are all employed by the innate immune system [1]. Recent study [2] has investigated the expression profiles of genes that might be crucial under stress. The expression of early stress response genes encoding immune-related molecules such as cytokines and toll-like receptors (TLRs) is one of many defensive processes that are induced to protect the cells of various tissues from heat-generated stress [2]. Toll-like receptors are crucial for initiating the adaptive immune response for long-term protection and for the innate immune response against bacteria, viruses, fungi, and parasites in the early stages of infection [3]. *Toll-like receptors 2* and *4* are involved in detecting components of the bacterial cell wall including lipopolysaccharide (LPS). *In vivo*, TLR2 and TLR4 can identify several bacterial cell wall components and TLR2 is crucial for recognizing Gram-positive bacteria [4]. Toll-like receptor gene expression was shown to occur in the peripheral blood mononuclear cells (PBMCs) of Thai indigenous chickens such as Betong chickens and to function in the immune response [5]. In broiler chickens under acute heat stress, *TLR* genes were suggested to play different roles in liver, kidney, spleen, heart, and small intestine by controlling the expression of *TLR4* mRNA [6]. The *LPS-induced tumor necrosis factor-α factor* (*LITAF*) is a crucial transcription factor that regulates the expression of genes involved in the production of inflammatory cytokines, apoptosis, and the immune system [7]. A quantitative real-time polymerase chain reaction (qRT-PCR) study showed that chicken *LITAF* mRNA was mostly expressed in spleen and intestine intraepithelial lymphocytes. The *LITAF* gene has been linked to the control of *TNF-α* gene expression during the development of coccidiosis or carcinogenesis [8, 9]. Four days after infection in older chickens, a rise in the number of macrophages in the cecal tonsils was shown to be associated with an increase in the level of *LITAF* gene expression. The *LITAF* gene plays a crucial role in lymphoid tissue pathogenesis and immune response [10].

Thai indigenous chicken (*Gallus gallus domesticus*) has a long history of domestication in rural Thailand and is crucial to low-income inhabitants of Thailand’s rural areas [11]. In Thailand, indigenous chicken meat has several distinctive qualities and appears to have certain benefits over that of imported breeds, particularly when produced for a niche market of consumers who prefer chewy and low-fat chicken meat [12]. The previous study revealed that the heterophil-to-lymphocyte ratio, abdominal exudative cell phagocytic activities, and serum anti-sheep red blood cell (RBC) titer were used to assess the ability of a Thai indigenous crossbred line to tolerate the tropical climate as well as its immune functions in terms of stress, and innate and humoral immune responses [13].

This study aimed to examine the hematological profile and *TLR2*, *TLR4*, and *LITAF* gene expression of PBMCs in Thai indigenous chickens.

## Materials and Methods

### Ethical approval

The study was approved by Kasetsart University Institutional Animal Care and Use Committee (ACKU65-AGK-026).

### Study period and location

The study was conducted from January 2022 to June 2022. All chickens were randomly collected from a chicken farm at Department of Animal Science, School of Agriculture and Natural Resources, University of Phayao, Thailand. The samples were processed at Department of Animal Science, Faculty of Agriculture, Kasetsart University.

### Animals and phenotypes

Three breeds of Thai indigenous chickens were included in this study: Black-boned chicken, Pradu Hang Dam chicken, and Fah Luang chicken (n = 5 per breed). Thai indigenous chickens were randomly selected from a chicken farm at the Department of Animal Science, School of Agriculture and Natural Resources, University of Phayao, Thailand. The chickens were 16 weeks of age. The samples (2 mL) were collected from wing vein in ethylenediaminetetraacetic acid-containing tubes to prevent blood coagulation.

### Blood hematological analysis

The hematological analyses including RBCs, hemoglobin, hematocrit, platelets, and white blood cells (WBCs) were performed using a hematological analyzer (Cell Dyn 3700, Wiesbaden Hesse, Germany).

### Peripheral blood mononuclear cell isolation

Peripheral blood mononuclear cells were isolated from the blood samples using a method described by Böyum [14]. Briefly, 2 mL of blood was gently added over 2 mL of Lymphoprep density gradient medium (Stemcell Technologies, Cologne, Germany) followed by centrifugation for 30 min at 277× *g*. The white band of mononuclear cells was observed and then collected for further analysis.

### mRNA expression of *TLR2, TLR4*, and *LITAF* genes

Total RNA was extracted from PBMCs of three chicken breeds (n = 5 per breed) using QIAamp RNA Mini Kit (Qiagen, Courtaboeuf, France). The purity of the extracted RNA was determined using a NanoDrop spectrophotometer, with A_260/280_ ratio ranging from 1.80 to 2.10. The real-time polymerase chain reaction was performed using a MyGo Pro^®^ real-time PCR instrument (IT-IS Life Science Ltd., Middlesbrough, UK) with a reaction mixture using QuantiNova SYBR Green RT-PCR Kit (Qiagen, Hilden, Germany), consisting of 10 μL of 2× QuantiNova SYBR Green RT-PCR Master Mix, 1 μL each of 10 μM (0.5 μM) forward and reverse primers, 0.2 μL of QN SYBR Green RT Mix, 5 μL of template, and 2.8 μL of nuclease-free water, made up to a total volume of 20 μL. A two-step amplification program was performed, involving pre-denaturation at 95°C for 2 min, followed by 40 cycles of denaturation at 95°C for 5 s, annealing at 60°C for 10 s, and extension at 72°C for 15 s, with the collection of fluorescence signal at the end of each cycle. Melting analysis was performed from 60°C to 97°C at 0.1°C/s. All samples were replicated technically, and the mean of the two replications was taken as the final result. The expression level was calculated from the ratio of the Cq value of the target gene to that of the housekeeping gene *ß-Actin*. The Primer3 software PCR was used to design PCR primers [15], as shown in Table-1.

**Table-1:** Primers sequence for *TLR2*, *TLR4*, *LITAF*, and *ß-actin* genes.

Gene	Primer sequence	Application	Tm (°C)	Product
*TLR2*	Fw: 5’- CTGATCCTGTGCCAATCAGA-3’	qRT-PCR	60	183 bp
	Rw: 5’- CCTGGTGCTCCATCTCAAGT-3’			
*TLR4*	Fw: 5’- ACAGGTGCCACATCCATACA-3’	qRT-PCR	60	222 bp
	Rw: 5’- TATGGCCCAGATTCAGCTCC-3’			
*LITAF*	Fw: 5’- CCTTGCAGTGGGACATCTCT-3’	qRT-PCR	60	190 bp
	Rw: 5’- GTGTCTTGGAATGAGCAGCA-3’			
*ß-actin*	Fw: 5’- CCACCGCAAATGCTTCTA-3’	qRT-PCR	60	96 bp
	Rw: 5’- GCCAATCTCGTCTTGTTTTATG-3’			

qRT-PCR=Quantitative real-time polymerase chain reaction, *TLR*=*Toll-like receptor*, *LITAF*=*Lipopolysaccharide-induced tumor necrosis factor-α factor*, Tm=Temperature

### Statistical analysis

The effects of chicken breeds on hematological parameters and gene expression were analyzed by analysis of variance in Statistical Analysis System (SAS version 9.2) (SAS Inst. Inc., Cary, NC, USA). Differences were considered to be statistically significant at p < 0.05. The results are presented as least squares means with the standard errors.

## Results

### Hematological parameters

The hematological parameters in the Thai indigenous chickens are presented in Table-2. Significant differences in RBCs, hemoglobin, and WBCs were observed (p < 0.05). Black-boned chicken and Fah Luang chicken had lower RBC levels than Pradu Hang Dam chicken (2.39 ± 0.24, 2.62 ± 0.16 vs. 3.53 ± 0.30 × 10^6^ cells/mm^3^, respectively). Fah Luang chicken had lower hemoglobin than Pradu Hang Dam chicken (14.58 ± 1.18 vs. 20.66 ± 2.10 g/dL). However, Fah Luang chicken had a higher WBC level than Pradu Hang Dam chicken (11858 ± 1062 vs. 6512 ± 982.90 cells/mm). There were no significant differences among the groups in terms of hematocrit, heterophils, basophils, eosinophils, lymphocytes, or monocytes (p > 0.05).

**Table-2:** Blood hematological parameters in Thai indigenous chickens.

Hematological parameters	A (n=5)	B (n=5)	C (n=5)	p-value
RBC (×10^6^ cells/mm^3^)	2.39±0.24^a^	2.62±0.16^a^	3.53±0.30^b^	0.013
Hemoglobin (g/dL)	16.40±1.01^ab^	14.58±1.18^a^	20.66±2.10^b^	0.039
Hematocrit (%)	36.80±2.48	37.80±3.43	49.20±5.01	0.072
WBC (cells/mm^3^)	9790±166.80^ab^	11858±1062^a^	6512±982.90^b^	0.043
Heterophils (%)	50.00±8.94	50.60±2.25	48.20±5.67	0.961
Basophils (%)	1.40±0.51	1.40±0.68	0.40±0.24	0.313
Eosinophils (%)	0.20±0.20	0.20±0.20	0±0	0.619
Lymphocytes (%)	46.40±9.43	45.80±2.06	49.20±5.54	0.924
Monocytes (%)	2.00±0.4472	2.00±0.54	2.20±0.49	0.948

A=Black-boned chicken, B=Fah Luang chicken, C=Pradu Hang Dam chicken, RBC=Red blood cell, WBC=White blood cell. ^a, b^Values within row with different superscripts differ significantly at p *<* 0.05

### Expression of *TLR2, TLR4,* and *LITAF mRNA*

**Figure-1:**
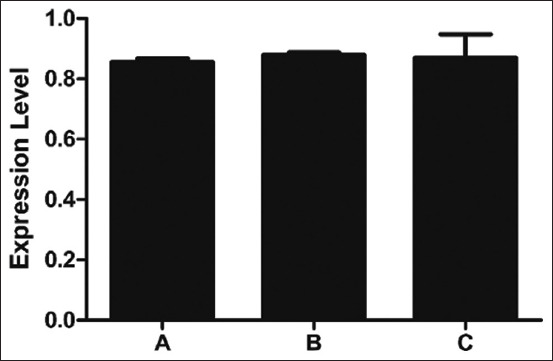
Normalized expression level of *toll-like receptor* (*TLR*) *2* gene transcript in Thai indigenous chickens (n = 5 per group). The expression level was expressed as the ratio of Cq values of *TLR2* gene with housekeeping gene *ß-actin*. A=Black-boned chicken, B=Fah Luang chicken, C=Pradu Hang Dam chicken.

**Figure-2:**
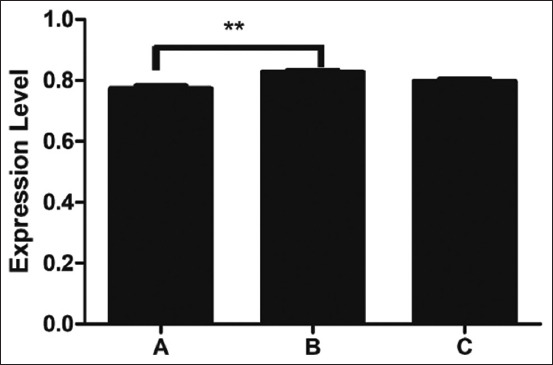
Normalized expression level of *toll-like receptor* (*TLR*) *4* gene transcript in Thai indigenous chickens (n = 5 per group). The expression level was expressed as the ratio of Cq values of *TLR4* gene with housekeeping gene *ß-actin*. A=Black-boned chicken, B=Fah Luang chicken, C=Pradu Hang Dam chicken.

**Figure-3:**
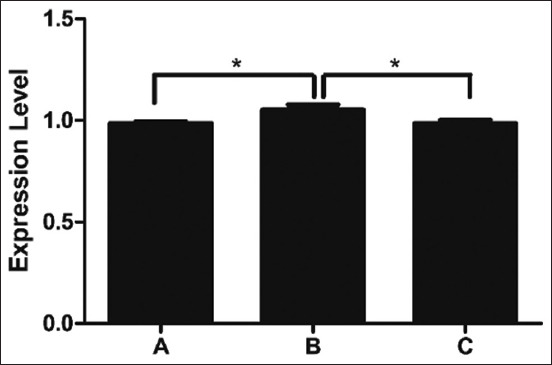
Normalized expression level of *lipopolysaccharide-induced tumor necrosis factor-α factor* (*LITAF*) gene transcript in Thai indigenous chickens (n = 5 per group). The expression level was expressed as the ratio of Cq values of *LITAF* gene with housekeeping gene *ß-actin*. A=Black-boned chicken, B=Fah Luang chicken, C=Pradu Hang Dam chicken.

This study revealed the genes abundantly expressed at the transcriptional level in three breeds of Thai indigenous chickens. The expression of *TLR2* mRNA did not differ significantly among the groups (p > 0.05) (Figure-1). The *TLR2* gene expression levels of black-boned chicken, Fah Luang chicken, and Pradu Hang Dam chicken were 0.856 ± 0.011, 0.879 ± 0.009, and 0.870 ± 0.078, respectively. The *TLR4* mRNA level differed significantly among the groups (p < 0.01) (Figure-2), with levels in black-boned chicken, Fah Luang chicken, and Pradu Hang Dam chicken of 0.774 ± 0.009, 0.827 ± 0.007, and 0.797 ± 0.009, respectively. The *LITAF* gene expression differed significantly among the groups (p < 0.05) (Figure-3), peaking in Fah Luang chicken at 1.054 ± 0.024, while the levels in black-boned chicken and Pradu Hang Dam chicken were 0.987 ± 0.006 and 0.986 ± 0.015, respectively.

## Discussion

Thai indigenous chickens are referred to as native species. There are numerous methods for enhancing economically important characteristics in chickens. Conventional and molecular breeding has successfully improved the genetics of Thai indigenous chickens by boosting growth, egg production, and climate change adaptation [16]. In this study, hematological parameters, including RBCs, hemoglobin, and WBCs were shown to differ significantly among Thai indigenous chicken breeds. Black-boned chicken and Fah Luang chicken had lower RBC levels than Pradu Hang Dam chicken. Fah Luang chicken had lower hemoglobin and higher WBC level than Pradu Hang Dam chicken. In a previous study, chicken blood before stress was shown to have RBCs of 3.25 ± 0.06 × 10^12^ cells/L, WBCs of 23.10 ± 1.02 × 10^9^ cells/L, and hemoglobin of 67.61 ± 2.49 g/L. Meanwhile, increases in hemoglobin content and RBC production were seen for 1 h after exposure to stress [17]. Moreover, birds with a restricted diet had fewer WBCs than birds fed *ad libitum* [18]. However, the present study as undertaken on healthy chickens. Hematological profiles show variability among different chicken breeds. The significant increase in WBCs may only serve to emphasize the importance of the local chicken in immunological health [11]. Age, sex, season, and nutrition have also been documented to affect hematological parameters in birds. It was reported that an increase in metabolic activity required to meet the energy demands under stress may be associated with an increase in hemoglobin concentration [19].

The expression of the *TLR2* gene did not differ significantly among the groups in this study. This means that the *TLR2* gene is not a major factor in the immune responses in Thai indigenous chickens. Moreover, mRNA expression of *TLR4* peaked in Fah Luang chicken. Toll-like receptors may exhibit different functions in the liver, kidney, spleen, heart, and small intestine of chickens under acute heat stress by controlling the expression of *TLR4* mRNA [6]. In one study, after exercise-induced stress, *TLR4* gene expression was found to be increased in both muscle tissue and WBCs [20]. However, this is an excellent opportunity to examine *LITAF* expression and its role in the avian inflammatory response. The mRNA expression of the *LITAF* gene peaked in Fah Luang chicken. In this study, this gene’s expression was correlated with the hematological parameters of WBCs, RBCs, and hemoglobin. The number of chicken macrophages also increased in correlation with the level of *LITAF* gene expression. The *LITAF* gene plays a crucial role in lymphoid tissue during pathogenesis and immune response [10]. The *LITAF* is also an important transcription factor that mediates the expression of inflammatory cytokines. *Lipopolysaccharide-induced tumor necrosis factor-α factor*gene expression is relatively high, indicating that it is important for preventing microbial infection [21]. The results of this study revealed that *TLR4* and *LITAF* genes might be the main genes involved in the immune response in Thai indigenous chickens.

## Conclusion

This study provided physiological data on healthy Thai indigenous chickens. The hematological parameters of RBCs, hemoglobin, and WBCs were shown to differ significantly among Thai indigenous chickens. The *TLR4* and *LITAF* genes were found to be expressed in the PBMCs of Thai indigenous chickens, with particularly higher levels found in Fah Luang chicken. It could be concluded that the *TLR4* and *LITAF* genes might be involved in the main immune responses in Thai indigenous chickens. Further study is required to determine the biological role of the *TLR* family of genes in immune function in chickens.

## Authors’ Contributions

AK and CT: Carried out the study, analyzed the data, and drafted the manuscript. AG, MJU, WN, CB, and WL: Provided technical help during the experiments. AK: Study conception, study design, and reviewed the manuscript. AK and CT: Revised the manuscript. All authors have read and approved the final manuscript.
